# Immunomodulatory Properties of HLA-G in Infectious Diseases

**DOI:** 10.1155/2014/298569

**Published:** 2014-04-15

**Authors:** Laurence Amiot, Nicolas Vu, Michel Samson

**Affiliations:** ^1^Institut National de la Santé et de la Recherche Médicale (Inserm), U.1085, Institut de Recherche sur la Santé, l'Environnement, et le Travail (IRSET), 2 Avenue du Pr. Leon Bernard CS 34317, 35043 Rennes, France; ^2^Université de Rennes 1, 35043 Rennes, France; ^3^Fédération de Recherche BioSit de Rennes UMS 3480, 35043 Rennes, France; ^4^Department of Biology, University Hospital Pontchaillou, CHU Pontchaillou, 35033 Rennes, France

## Abstract

HLA-G is a nonclassical major histocompatibility complex molecule first described at the maternal-fetal interface, on extravillous cytotrophoblasts. Its expression is restricted to some tissues in normal conditions but increases strongly in pathological conditions. The expression of this molecule has been studied in detail in cancers and is now also beginning to be described in infectious diseases. The relevance of studies on HLA-G expression lies in the well known inhibitory effect of this molecule on all cell types involved in innate and adaptive immunity, favoring escape from immune control. In this review, we summarize the features of HLA-G expression by type of infections (i.e, bacterial, viral, or parasitic) detailing the state of knowledge for each pathogenic agent. The polymorphism, the interference of viral proteins with HLA-G intracellular trafficking, and various cytokines have been described to modulate HLA-G expression during infections. We also discuss the cellular source of HLA-G, according to the type of infection and the potential role of HLA-G. New therapeutic approaches based on synthetic HLA-G-derived proteins or antibodies are emerging in mouse models of cancer or transplantation, and these new therapeutic tools may eventually prove useful for the treatment of infectious diseases.

## 1. Introduction

HLA-G was first described by Geraghty et al. in 1987 [1] as a member of the nonclassical human leukocyte antigen (HLA) family, which also includes HLA-E and F [2, 3]. The HLA-G gene is located within the major histocompatibility complex on the p21.31 region of chromosome 6. It has eight exons and seven introns, and its sequence is about 86% identical to the consensus sequence of the HLA-A, -B, and -C genes. Unlike classical class I molecules, HLA-G has a short cytoplasmic tail of six amino acids, due to premature stop codon in exon 6 [1]. Alternative splicing of the primary transcript generates four membrane-bound isoforms and three soluble forms. HLA-G1 has a structure similar to that of classical HLA class I molecules: a heavy chain consisting of three extracellular globular domains (*α*1, *α*2, *α*3) noncovalently associated with the *β*-2 microglobulin and a monomer peptide. The membrane-bound isoforms, HLA-G2, -G3, and -G4, are truncated isoforms lacking the *α*2 and/or *α*3 domains of the heavy chain [4, 5] and they should not, therefore, bind *β*-2 microglobulin [6]. Soluble HLA-G isoforms are generated either by alternative splicing of the HLA-G primary transcript (HLA-G5, -G6, and -G7) or by proteolysis of the HLA-G1 isoform (HLA-G1s) [7–9]. Indeed, the HLA-G5, -G6, and -G7 isoforms are highly unusual, as they are spliced variants of the HLA-G mRNA retaining introns 4 and 2 [7, 9, 10].

HLA-G is structurally diverse, with (i) different isoforms resulting from alternative splicing, (ii) some *β*2 M-free molecules [11], and (iii) all isoforms other than HLA-G3 being able to form homomultimers [12]. Indeed, HLA-G isoforms can also form homotrimers and homodimers, through the establishment of disulfide bridges between cysteine residues located in positions 42 and 147 [13]. Truncated isoforms of HLA-G can also carry out the biological functions of this molecule. Indeed, multimeric structures of HLA-G isoforms function by differential binding to LILRB receptors [12]. Thus, HLA-G has specific features not found in other HLA class I molecules, such as (i) limited polymorphism [14, 15], (ii) restricted expression in physiological conditions [16], (iii) a shorter cytoplasmic tail region due to a stop codon in exon 6, (iv) unusual regulatory mechanisms due to the use of a promoter unique among HLA class I genes [17–20], and (v) numerous immunomodulatory properties, as described below.

HLA-G expression was initially described as restricted to the maternal-fetal interface, on extravillous cytotrophoblasts [21]. In healthy, nonfetal subjects, the HLA-G protein is found only on the cornea [22], thymic medulla [23], nail matrix [24], beta cells of the islets of Langerhans [25], mesenchymal stem cells [26], and endothelial precursors [27].

Levels of this protein are upregulated in many diseases and this upregulation may modulate the immune response.

**Figure 1:**
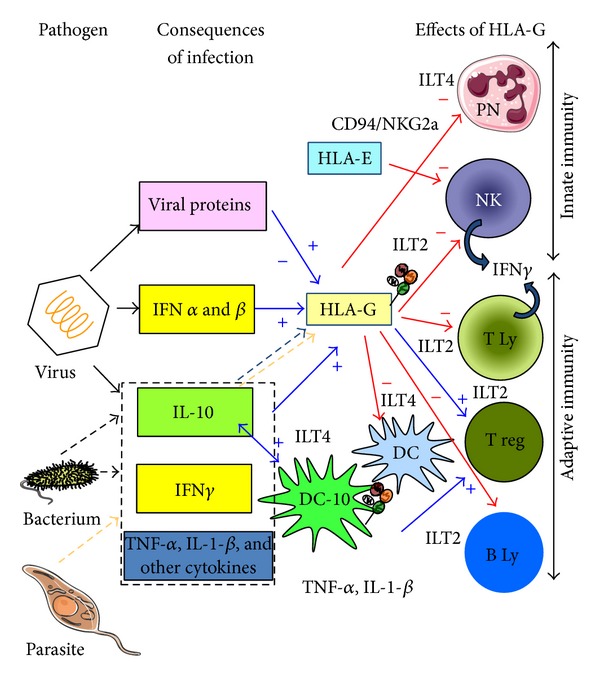
Causes and consequences of HLA-G modulation in infectious diseases. Positive and negative effects of HLA-G are shown in blue and red, respectively. Parasites, bacteria, or viruses induce the secretion of various cytokines, including IL-10 and interferon (-*γ* for bacterium and IFN-*α* and -*β* for virus). These cytokines upregulate the expression or secretion of HLA-G. In addition, IL-10 induces IL-10-producing human dendritic cells (DCs), termed DC-10, expressing HLA-G and ILT4. HLA-G induces tolerogenic DC in addition to DC-10 and regulatory cells via direct interaction with ILT2 and/or ILT4. HLA-G, through direct interaction with ILT2, inhibits the function of T and NK cells and B cells, whereas it inhibits the function of granulocytes and myeloid DC via direct interaction with ILT4. Indirect effects of HLA-G are mediated by the induction of HLA-E cell surface expression, which inhibits CD94/NKG2a on NK and T cells. The consequence of HLA-G action is a downregulation of innate and adaptive immunity.

The immunosuppressive properties of HLA-G have been thoroughly described. Indeed, the role of this molecule in immunotolerance was first described following its detection at the maternal-fetal interface, in* in vitro* studies, and has recently been confirmed by* in vivo* studies in mice. HLA-G can inhibit all types of immune competent cells (Figure 1). This effect is mediated by the direct binding of both completely soluble and membrane-bound isoforms to inhibitory receptors via the *α*3 domain. Indeed, B and T lymphocytes, NK cells, and monocytes of the myeloid lineage express the immunoglobulin-like transcript ILT2 (CD85j, ILIRB1) [28]; monocytes, macrophages, and dendritic cells express ILT-4 (CD85d, LILRB2) [29]. The killer cell immunoglobulin-like receptor (KIR2DL4/p49) is specific for HLA-G and is expressed by decidual NK cells. Unlike other inhibitory receptors, it may also mediate activation [30, 31]. In addition, soluble HLA-G triggers the apoptosis of T and NK cells via CD8-like classical class I soluble molecules [32]. HLA-G modulates adaptive and innate immunity by interacting with T or B lymphocytes and NK cells or polymorphonuclear cells (Figure 1).

HLA-G can inhibit all steps in the immune response: differentiation, proliferation, cytolysis, cytokine secretion, and immunoglobulin production. It can also alter antigen presentation to T lymphocytes, by inhibiting dendritic cell function and maturation [33–36] and by specific effects on T and B lymphocytes during effector activities. Indeed, this molecule inhibits the cytolytic activity of T and NK cells [37, 38] and the proliferation of B lymphocytes, together with the differentiation of these cells and their immunoglobulin secretion [39]. It also affects cooperation between B and T lymphocytes, by inhibiting T4 alloproliferation [36, 40] and inducing different types of regulatory T cells [41, 42]. Trogocytosis can generate different types of temporary regulatory cells* in situ*, accounting for the immunosuppressive effect of HLA-G-positive cells, despite their small numbers [43]. In addition, HLA-G inhibits the function of neutrophils, key cells in host immune defense against pathogens. Indeed, its interaction with its receptor, ILT4, on neutrophils impairs phagocytosis and the respiratory burst of neutrophils responsible for reactive oxygen species production [44].

Many studies have focused on HLA-G in tumoral processes, highlighting its role in tumor escape from the immune response [45]. The expression of this molecule is also beginning to be reported in other diseases, including infectious diseases. As host immune defense mechanisms efficiently eliminate most infections, studies on HLA-G in infections are based on the rationale that this molecule decreases the efficacy of the immune response through wide-ranging effects on all cell types involved in the immune response.

## 2. Features of HLA-G Expression by Infection Type

The main studies on HLA-G and infectious diseases are summarized in Table 1.

*(a) Bacterial Infections. *Septic shock is characterized by high mortality (40–50%) despite adequate initial treatment. Indeed, during septic shock, the initial huge systemic inflammatory response is immediately followed by an anti-inflammatory process, acting as negative feedback. However, this compensatory inhibitory response may subsequently become deleterious, as nearly all immune functions are compromised [46].

Monneret et al. [47] reported that marked, persistent HLA-G5 expression in septic shock was predictive of survival. The exocytosis-mediated upregulation of ILT4 expression on neutrophils is inhibited in conditions of sepsis, so the large amounts of HLA-G5 found in the plasma samples of patients surviving sepsis may have allowed them to control neutrophil inflammatory activity [44]. However, soluble HLA-G concentration was not found to be predictive of the detection of bacteremia and sepsis in pediatric oncology patients with chemotherapy-induced febrile neutropenia [48]. 

*(b) Parasitic Infections.* Few clinical data for parasitic infections are available, and those published relate mostly to plasma concentrations of sHLA-G, with the exception of one study of the protective role of HLA-G polymorphism in malaria [49]. We previously reported an increase in soluble HLA-G levels in 35% of cases of visceral leishmaniasis (*Leishmania infantum*) (VL) in HIV-seronegative patients and 57% of patients coinfected with HIV and* Leishmania infantum* [50]. However, the percentage of HLA-G-positive patients and the mean sHLA-G value were significantly lower in patients with both HIV infection and VL than in the patients with HIV infection alone. These results suggest that the increase in sHLA-G levels in HIV-infected patients with VL may contribute to a general tolerogenic environment, favoring the persistence of* Leishmania* and shortening the life expectancy of HIV-infected patients. sHLA-G may also be an immune biomarker of successful treatment. Thus, levels of sHLA-G with indoleamine 2,3 dioxygenase (IDO) activity may thus constitute, together with Th1/Th2 cytokine levels, surrogate markers for the resolution of VL, at least in immunocompetent patients [51]. High levels of sHLA-G are found in the amniotic fluid in women acquiring toxoplasmosis during pregnancy. The levels of this protein are the highest when the fetus is congenitally infected. However, all fetuses were born alive in our small series of patients, consistent with adequate downregulation of the inflammatory response. HLA-G may, therefore, play an immunomodulatory role that is necessary to avoid fetal loss but that may lead to the maternal-fetal transmission of* Toxoplasma gondii* [52]. HLA-G expression increases upon the* in vitro* infection of primary human trophoblasts and BeWo cells with* Toxoplasma gondii*, probably due to the secretion of proinflammatory cytokines in response to the parasite [53]. 

*(c) Viral Infections.* Many extensive studies have been carried out on cancers, but HLA-G expression has also been studied in many viral infections, with HIV infections being the most extensively studied (at least 30 published studies).

### 2.1. HIV Infection

Levels of sHLA-G are significantly higher in HIV-infected patients before treatment than in healthy controls [54]. The increase in plasma sHLA-G concentration in these patients has been attributed to an increase in HLA-G secretion from intracellular stores in monocytes and dendritic cells [55]. Indeed, a longitudinal study of plasma sHLA-G concentration in HIV-infected individuals with different rates of clinical progression showed that sHLA-G expression was associated with HIV disease progression [56]. HLA-G levels are high early in infection and remain high in rapid progressors. However, these concentrations return to normal levels in the chronic phase of infection, in both untreated normal progressors and long-term nonprogressors, when the infection is controlled. Cell surface expression of HLA-G is also detected on 93% of monocytes and 34% of T lymphocytes in patients [57]. Serum concentrations of HLA-G, like those of the other classical class I molecules (sHLA-A, -B, -C), also increase in HIV-infected patients and are significantly decreased by antiretroviral therapy (highly active antiretroviral therapy, or HAART), in cases in which HIV-1 replication is strongly inhibited.

Moreover, HAART significantly decreases the concentration of circulating soluble HLA-G molecules, this decrease being correlated with viral clearance and an increase in CD4^+^ T cells, as reported for classical class I molecules. The decrease in sHLA-G levels after HAART reported by Cabello et al. is not consistent with the finding of an increase in HLA-G expression on monocytes following HAART in another study [56]. The agents responsible for this increase are nucleoside reverse transcriptase inhibitors rather than protease inhibitors [58]. Murdaca et al. explain these conflicting findings in terms of the membrane expression of HLA-G inducing an increase in soluble HLA-G molecule shedding [59]. However, high levels of HLA-G in peripheral monocytes were also observed in two of the 12 untreated patients, suggesting other causes unrelated to HAART [56]. High levels of HLA-G molecules are also found in the monocytes of untreated HIV-positive patients [57], possibly due to the pathogenesis of infection.

HLA-G expression may allow these cells to evade the immune system, because the protective function of HLA-G occurs after the induction of this molecule in HAART-treated HIV-1 patients, accounting for both the consistently defective function of monocytes in HIV-1-infected patients and the role of the viral reservoir present in monocytes during infection [56]. It inhibits myeloid dendritic antigen-presenting capacity via ILT4 and enhances the secretion of inflammatory cytokines [55]. HLA-G^+^ regulatory T cells decrease in both absolute numbers and relative proportions during progressive HIV-1 infection. Their levels are thus inversely correlated to those of phenotypic markers of immune activation. HLA-G^+^ T regulatory cells can decrease harmful bystander activation and may protect against HIV-1-associated immune activation and HIV-1 disease progression [60].

### 2.2. Human CMV (hCMV)

Both membrane-bound and soluble plasma HLA-G concentrations increase during hCMV infection. The induction of HLA-G protein in macrophages has been observed after the generation of these cells* ex vivo* from latently infected monocytes and after the reactivation of hCMV infection [61]. HLA-G protein has also been detected* ex vivo* on bronchoalveolar macrophages from patients suffering from acute hCMV pneumonitis, on peripheral monocytes and in plasma [62]. Blood sHLA-G concentration has been shown to be correlated with blood IL-10 and IFN-*γ* concentrations.

### 2.3. Neurotropic Virus

HLA-G protein has been reported to be expressed in human neurons after infection with rabies virus or herpes simplex type I, following the activation of gene transcription [63, 64].

### 2.4. Influenza A Virus (IAV)

HLA-G expression was first demonstrated* in vitro* in an alveolar epithelial cell line, at the mRNA and protein levels, after treatment with various IAV strains [65]. HLA-G expression has been detected* in vivo* in patients infected with the pandemic H1N1 or seasonal H1N1 [66] viruses. It has been detected on monocytes and T lymphocytes, including T4 regulatory cells in particular. This cellular HLA-G expression contrasts with the absence of an increase in the plasma concentration of this protein.

### 2.5. Human Papilloma Virus (HPV)

Low levels of HLA-G5 expression are observed in all HPV-related cases of invasive cervical cancer [67]. Indeed, HPV E5 may be involved in the decrease in HLA-G expression at the cell surface, because high-risk HPV oncoproteins may inhibit the promoters of HLA class I heavy chain genes and may modulate the levels of the transporter associated with antigen processing (TAP1) protein.

### 2.6. Hepatitis B and C Viruses (HBV and HCV)

Plasma HLA-G concentration is higher during hepatitis infection than in healthy subjects without HBV infection. It is higher in cases of chronic hepatitis B than in acute hepatitis B and it returns to normal after resolution of the infection. In addition, an increase in HLA-G cell surface expression is observed on peripheral monocytes and regulatory T cells [68]. Similarly, an increase in blood sHLA-G concentration has been reported in patients with chronic hepatitis infection [69], associated with an increase in blood IL-10 and IFN-*γ* concentrations.

HLA-G expression in the liver has been detected by immunohistochemical methods, in hepatocytes and biliary epithelial cells from patients with chronic hepatitis B, by Souto et al. [70]. We [71] found that the number of HLA-G^+^ cells was significantly correlated with the area of tissue affected by fibrosis. This led to the first demonstration that HLA-G^+^ cells were mast cells. HLA-G secretion was significantly induced in human mast cells stimulated with IL-10 or class I interferons.

## 3. Mechanisms of HLA-G Modulation during Infection

These mechanisms (polymorphism, interference of infectious proteins with HLA-G intracellular trafficking and shedding, and cytokines) are summarized in Figure 1 and Tables 2 and 3.

*(a) Polymorphism, Alleles, and Single-Nucleotide Polymorphisms.* Firstly, HLA-G polymorphism, although limited with 40 alleles identified [15], is involved in susceptibility to viral infections, particularly those caused by HIV and HCV (Table 1). Indeed, the G*010108 allele has been reported to be associated with an increase in the risk of HIV-1 infection, whereas the G*0105N allele (null allele) has been shown to be associated with protection from infection in African women [72, 73] but a greater risk of infection in a population from north-eastern Italy [74]. Da Silva et al. have shown that HLA-G variants influence the horizontal transmission of HIV horizontal in African-derived HIV-infected patients, with a higher frequency of alleles and genotypes associated with low levels of HLA-G expression (i.e., a higher frequency of the 14 bp insertion allele) in African-derived HIV-infected individuals and a higher frequency of the 14 bp insertion +3142G (insG) haplotype and the insG/insG diplotype. In addition, a higher frequency of the ins/ins genotype is found among African-derived HIV-infected patients also infected with HCV [75].

Thus, the transmission of HIV-1 from infected mothers to their infants may be influenced by dissimilarities in their HLA-G sequences [76]. HLA-G*01:03+ mothers have recently been shown to be less likely to transmit HIV-1 to their children during the perinatal period [77]. The polymorphic sites may affect miRNA binding to the HLA-G mRNA, thereby influencing HLA-G translation [19, 78].

HLA-G polymorphism may also affect susceptibility to HCV infection in patients with sickle cell disease, because the C allele seems to confer protection against HCV, by a mechanism associated with an increase in HLA-G expression [79]. Homozygosity for the 14 bp deletion and the allele containing this deletion (010401) seems to be a risk factor for the vertical transmission of HCV, whereas the 0105N allele confers protection [80]. The HLA-G 14 bp insertion/deletion polymorphism is also a putative susceptibility factor for active hCMV infection in children [81]. Two polymorphisms in the 3′ untranslated region of the HLA-G gene (3′UTR) (14 bp ins/del, +3142C>G) are involved in susceptibility to HPV infection; indeed, the 14 bp del allele is associated with a high risk of HPV infection, and the del/C haplotype is associated with the development of invasive cervical cancer [82].

An association of HLA-G 3′UTR polymorphisms with the antibody response to* Plasmodium falciparum* has also recently been reported [49, 83].

*(b) Interference of Viral Proteins with the Intracellular Trafficking of HLA-G.* Viral proteins have generally been reported to decrease HLA class I expression, but their effect on HLA-G expression at the cell surface is more ambiguous (Table 3). Indeed, they may have no effect [84–86] or an inhibitory effect [87–90], and one study, carried out by Onno et al. [61], even reported HLA-G induction after viral reactivation in activated macrophages, through the cooperative action of the early HCMV proteins pp72 and pp86. By contrast, another team showed that HLA-G1 levels at the cell surface were downregulated and that this downregulation was dependent on hCMV short viral US glycoproteins [89]. Some US proteins have differential effects on the expression of classical HLA class I and HLA-G molecules at the cell surface, due to the shorter cytoplasmic tail of HLA-G [91] and other structural characteristics [91].

These conflicting results for hCMV may be accounted for by differences between the cell types studied (monocytes, trophoblasts, or the U373-MG astrocytoma cell line). The effects of viral proteins differ with the infected cell target, the type (classical or otherwise) of HLA class I molecules, and the membrane-bound or soluble nature of the HLA-G protein. These conclusions are illustrated by the following examples. US10 downregulates the cell surface expression of HLA-G but not that of classical class I MHC molecules [88], because the short cytoplasmic tail of HLA-G (RKKSSD) acts as a US10 substrate. On the other hand, the US2 protein decreases levels of HLA class I molecules by supporting proteasome-mediated degradation, unlike HLA-G1, which lacks the residues essential for interaction with US2 [84]. Moreover, HLA-G1 has also been reported to be targeted for degradation, independently of the cytoplasmic tail [84].

For HIV infections, the short cytoplasmic tail of HLA-G confers resistance to Nef-induced downregulation [85], whereas Nef downregulates MHC class I molecules [92].

*(c) Cytokines.* Many viruses have also developed other strategies for escaping host immune surveillance, such as a deregulation of the host cytokine network through the secretion of cytokines. Cytokines are also important in bacterial infections.

The interleukin- (IL-) 10 family of cytokines and the related interferon (IFN) family form the larger class II cytokine family [93]. The IL-10 family consists of three subgroups, defined on the basis of biological functions: IL-10, the IL-20 subfamily cytokines (including IL-19, IL-20, IL-22, IL-24, and IL-26), and the type III IFN group (IFN*λ*s).

Several viruses have been shown to upregulate the expression of cellular IL-10, which is produced by monocytes and, to a letter extent, by lymphocytes and, possibly, mast cells. Other viruses, such as the Epstein-Barr virus and HCMV, have functional orthologs of IL-10. Indeed, blood IL-10 and IFN-*γ* concentrations are high in hCMV infection [62] and in chronic hCMV infection [69]. In bacterial infections, IL-10 is also produced during sepsis [94]. High IL-10 levels are associated with bacteremia and sepsis in febrile pediatric cancer patients with neutropenia [95].

IL-10 is a pleiotropic cytokine with both immunostimulatory and immunosuppressive properties [96]. HLA-G expression is induced following IL-10 stimulation in experiments* in vitro* and is associated with IL-10 expression* in vivo* in a context of cancer. IL-10 selectively induces HLA-G expression, at both the mRNA and protein levels, in human trophoblasts and monocytes [97]. By contrast, Zhao et al. [53] have reported that IL-10 downregulates HLA-G expression in an* in vitro* model based on the infection of human trophoblasts with* Toxoplasma gondii*.

Interferons trigger important antiviral effects during viral infections. They can be classified into three classes: (i) class I (IFN-*α*, -*β*), produced by NK cells, lymphocytes, macrophages and fibroblasts, and other molecules, such as IFN-*ω* and -*ζ*, produced by leukocytes, (ii) class II, consisting solely in IFN-*γ* produced by NK and T cells, and (iii) class III, recently described and including IFN-*λ*1 (IL-29), -*λ*2 (IL-28A), and -*λ*3 (IL-28B), produced by numerous cell types, including plasmacytoid dendritic cells. Types I and III interferons are produced by virus-infected cells. In these cells, double-stranded RNA activates the signaling cascades leading to the transcription of the IFN-*α* and -*β* genes. Following their secretion, these interferons interact with a specific IFN*α*/*β* receptor on neighboring uninfected cells and on the initial infected cells, activating a signaling cascade that produces antiviral proteins that act on viruses and upregulate HLA class I expression. IFN-*γ* is involved in both innate and adaptive immunity. Type III IFNs signal through a receptor complex consisting of IL10R2 and IFNL-R1 (IL-28RA). HLA-G induction by interferons has been reported in numerous studies. Indeed, different types of IFN (*α*, *β*, and *γ*) can induce HLA-G expression in different cell types. Yang et al. [98] reported the induction of HLA-G on Jeg 3 cells by different interferons. The induction of HLA-G on monocytes has also been reported [99]. IFN-*β* and -*γ* have recently been shown to activate HLA-G expression in a human neuron cell line infected with rabies virus [63]. HLA-G expression after IFN treatment has also been demonstrated in several tumor models, including a melanoma cell line. Thus, treatment with IFN-*β* or -*γ* increases the dimer/monomer ratio and, subsequently, affinity for the ILT2 receptor [100]. An increase in HLA-G expression, in monocytes and serum, is also observed in patients treated systemically with IFN-*α* [101]. Similar effects have also been reported after treatment with IFN-*β*1 [102]. Interferons are known to induce HLA class I expression by binding to the interferon-stimulated response element (IRSE) motif in the proximal promoter region of class I genes. This motif is absent from the HLA-G promoter [15], so the upregulation of HLA-G expression by interferons was unexpected. This upregulation was accounted for by the identification of another specific functional IRSE in the distal promoter, at a position −744 bp upstream from the ATG [103].

The early phase of septic shock is characterized by a massive release of inflammatory mediators, causing organ dysfunction and hypoperfusion. These cytokines include tumor necrosis factor-alpha (TNF-alpha), interleukin-1beta (IL-1beta), and IFN-*γ*. Like IFN-*γ*, TNF-*α* and IL-1 can also induce HLA-G. Indeed, TNF-*α* has been shown to induce a moderate increase in steady-state levels of HLA-G mRNA in human trophoblast cell lines [98]. IL-1*β* increases the expression of HLA-G and Toll-like receptor 4 (TLR4) in an HIF-1*α*-dependent manner [104].

Protease levels generally increase during bacterial and viral infections and this may lead to the proteolytic shedding of membrane-bound HLA-G in a soluble form, resulting in an increase in blood HLA-G concentration.

## 4. General Discussion

An upregulation of HLA-G expression has been reported in most studies of viral infection. Reported discrepancies in the results concerning HLA-G expression in hCMV or HIV infections may reflect differences in the models used or in infection status or stage between studies. This upregulation of HLA-G expression results principally from an increase in the secretion of cytokines, such as IL-10 and class I interferons. HLA-G levels increase, either at the cell surface or in the blood (sHLA-G). Indeed, concentrations of soluble HLA-G in the blood increase in some viral infections caused by HIV, hCMV, HCV, and HBV viruses, similar to classical soluble class I antigens. The increase in the secretion of cytokines, including interferons in particular, during the course of viral infection, and the use of interferons as therapeutic agents may account for the increase in HLA-G levels. Shedding, due to metalloprotease digestion, is favored by interferons and also contributes to the increase in soluble HLA-G concentration in the blood. The peripheral cells expressing HLA-G during viral infections are monocytes and T lymphocytes (HIV, influenza). Neurons and bronchoalveolar macrophages have been shown to express HLA-G in infected tissues. In HCV hepatitis, Souto et al. [70] found that hepatocytes and biliary epithelial cells expressed HLA-G, whereas we identified HLA-G-positive cells as mast cells [71]. This discrepancy can also be accounted for a difference in the definition of positivity, because we also observed a weak staining of hepatocytes but took only strong staining into account. These findings were confirmed by our findings for a human mast cell line showing that this cell line expressed HLA-G and secreted class I interferons. Moreover, mast cells may promote liver fibrosis [105] by stimulating collagen synthesis and fibroblast chemotaxis. Cytokines involved in liver fibrosis, such as IL-4 or IL-33 [106], act as chemoattractants, driving the activation of mast cells [107, 108]. In addition, mast cells secrete tryptase and many cytokines involved in fibroblast proliferation [109] and fibrogenesis [110], including IL-10 [111]. However, the role of HLA-G in viral infections remains unclear, because two hypotheses are possible. It may promote virus immune escape, as in cancers. This hypothesis is supported by the immunosuppressive properties of HLA-G, which act on all the cells involved in the immune response. In addition, sHLA-G downregulates CXCR3 levels on peripheral blood and tonsil CD56 cells [112]. This dysregulation of CXCR3 signaling due to CXCL10 deficiency impairs antiviral responses* in vivo, *including the antiviral response to herpes simplex virus 1 infection [113].

Alternatively, HLA-G expression or secretion may reflect an appropriate and efficient response to the inflammatory process occurring during viral infection or septic shock. Indeed, HLA-G may be beneficial during viral infection, because an increase in HLA-G concentration occurs following the secretion or therapeutic administration of interferons, classes I and III IFNs are secreted as physiologic antiviral responses, and IFN-*α* is an effective treatment for chronic HCV infection. We can hypothesize that the antiviral effect of classes I and III IFNs may be mediated by the properties of HLA-G, which is induced by IFN, as described above.

The immunosuppressive properties of HLA-G have been clearly demonstrated* in vitro*, and the role of this protein has now been elucidated* in vivo*. Indeed, two studies have demonstrated the involvement of this protein in tumor progression in a mouse model* in vivo*. In a xenograft model, the HLA-G1 isoform promotes tumor progression in immunocompetent Balb/c mice, affecting both innate and adaptive immunity. By contrast, no tumor development is observed when HLA-G is blocked by a specific antibody, demonstrating the specificity of the effect [114]. HLA-G plays a role in tumor escape, through expansion of the population of myeloid-derived suppressor cells and an alteration of the cytokine balance in favor of a Th2 response rather than a Th1/Th17 response. HLA-G expression is associated with tumor metastasis and poor survival in the Balb/c nu/nu mouse model of ovarian cancer [115]. In another model used to assess the efficacy of synthetic HLA-G proteins for therapeutic purposes in a context of transplantation, it was shown that a single treatment of skin allograft recipient mice with these proteins was sufficient to prolong graft survival significantly and that four weekly treatments were sufficient to ensure graft survival [116].

The feasibility of synthesizing effective HLA-G-derived molecules opens up new possibilities in the fields of tumor diseases and infection. For example, HCV infections are a worldwide public health problem and may be suitable for treatment with such molecules, because HLA-G expression is correlated with the area of fibrosis.

In the future, it may be possible to modulate HLA-G transcription with a miRNA, such as the hsa mir-148a and mir-152, which bind to the 3′ untranslated region of the HLA-G gene (3′UTR) [19], downregulating its mRNA levels. Indeed a polymorphism of the binding site for this miRNA (the 263del/ins SNP) has been associated with poor control of HIV infection [117].

## 5. Conclusions

As in cancers, HLA-G expression is upregulated in infectious diseases, in response to changes in the cytokine microenvironment, relating principally to increases in the levels of IL-10 and interferons. HLA-G expression may occur in infected tissues and/or, more frequently, in peripheral blood, in the form of sHLA-G or a membrane-bound form on monocytes or different types of T cells (CD4, T reg). This molecule may have deleterious effects, promoting pathogen escape from immune control, as reported in cancers, or it may be beneficial, as in septic shock [47], reflecting appropriate and effective feedback control of inflammatory process. The role of this protein in parasitic and viral infections remains to be elucidated. Thus, HLA-G may be a single marker of infectious diseases, related to pathogens and/or to the immune response, or it may constitute a therapeutic target, once its function has been clarified in particular types of infections.

## Figures and Tables

**Table 1:** Summary of the main studies on HLA-G and infectious diseases.

	HLA-G level changes			Model or patients	Characteristics	References
	Cell surface	Cell surface	Blood sHLA-G			
	Increase	Decrease	Increase			
*Infectious nonviral diseases *						
Septic shock			*↗*	*n* = 64	Marked early and persistent increase: predictor of survival	[47]

Parasitic infections due to *Plasmodium falciparum *						
		*↘*		Malaria-infected placenta (*n* = 15)	Extravillous trophoblast cells (42% versus 90% in controls) Association with an increase in NK cells	[118]

*Leishmania infantum* visceral leishmania (VL)			*↗*	*n* = 94 31 HIV+ 24 VL (7 HIV+ and 17 HIV−) 39 healthy subjects	HIV and VL: 57% HIV alone: 81% VL-HIV seronegative: 35%	[50]

*Toxoplasma gondii *	*↗*			*In vitro* infection of human trophoblast and BeWo cells	At mRNA and protein levels. Treatment with IL-10 decreases HLA-G expression.	[53]
			*↗*	Amniotic fluid: 58 women infected 24 noninfected	Significantly higher levels when the fetus is congenitally infected.	[52]

*Viral infections *						
HIV		*↘*		Cotransfection experiments on glioma cell line and macrophages	Nef-independent, Vpu-dependent	[119]
			*↗*		Before HAART, correlated with viral clearance and increase in CD4^+^ T-cell levels. Decrease after treatment (36 months) Role of interferons and cytokines Increase in shedding	[54]
	*↗*			Infection treated (20) or not treated (3)	Indirect induction by viral products and/or cytokines (IL-10) T lymphocytes and monocytes	[57]
	*↗*			Treated by HAART (*n* = 12)	Monocytes in treated patients Expression on monocytes decreases after treatment to block cytokines (IL-10)	[56]
	*↗*			Patients treated (*n* = 7) with HAART or with a protease inhibitor regime or after HAART stopped	Monocytes (50%) on HAART Increase with nucleoside reverse transcriptase inhibitors but not with protease inhibitors Decrease when HAART removed	[58]
			↗	Longitudinal study in 24 infected patients	In early phases, restored to normal level in chronic phases of untreated normal progressors and long-term nonprogressors Secretion by monocytes, dendritic cells Role of IL-10	[120]

hCMV	*↗*			hCMV reactivation in *in vitro* activated macrophages (*n* = 10) Patients with HCMV pneumonitis	Day 20 poststimulation: expression in 45% of macrophages Bronchoalveolar macrophages Cooperative action of pp72 and pp86	[61]
	*↗*		*↗*	Patients (*n* = 75)	Increase on peripheral monocytes (6.3% versus 1.6%) Association with increase in plasma IL-10 concentration and no significant increase in IFN-*γ* concentration	[62]

Neurotropic virus (HSV-1 and RABV)	*↗*			*In vitro* infection of human neuron cell line (NT2-N)	Activation of HLA-G transcription Cell surface expression during RABV infection but not during HSV-1 infection	[63, 64]

Influenza virus (IAV) H1N1	*↗*			*In vitro* IAV infection of human alveolar epithelial cell line A549	Upregulation of HLA-G m RNA and proteins	[65]
	*↗*			(101) HIN1 patients (58 pandemic and 43 seasonal H1N1)	Monocytes and T lymphocytes (T reg CD4CD25FOXP3)	[66]

HPV		*↘*		Biopsies of invasive cervical carcinoma (*n* = 79)	Low HLA-G5 expression in all HPV-related cases	[67]

Hepatitis B virus	*↗*		*↗*	90 acute, 131 chronic, and 152 resolved cases of hepatitis B	Chronic > acute Resolved = normal Expression on monocytes and Treg	[68]
	*↗*			Chronic hepatitis B (*n* = 74)	Hepatocytes and biliary epithelial cells	[70]

Hepatitis C virus			*↗*	Chronic hepatitis C (*n* = 67)	sHLA-G = sHLA-G1 and-G5 Increase in plasma IL-10 and IFN-*γ* concentrations	[69]
	*↗*			Liver biopsies of patients with chronic hepatitis C (*n* = 89)	Hepatocytes More frequent in milder stages	[121]
	*↗*			Liver biopsies of patients with chronic hepatitis C (*n* = 20)	Significant correlation with the area of liver fibrosis HLA-G-positive cells are mast cells Soluble HLA-G secretion by human mast cells regulated by class I interferons	[71]

**Table 2:** Influence of HLA-G polymorphism on susceptibility to infectious diseases.

Pathogens	Protection	Susceptibility	Vertical transmission (mother-to-child)	References
HIV	HLA-G*0105N (null allele)			[72, 73]
		G*010108 allele G*010108/010401 G*010101/010108		[72]
		G*0105N 14 bp (ins) allele +3142G (insG) haplotype		[74] [75] [75]
	G*01:01:01 genotype	G*01:04:04 genotype		[122]
			Differences in the HLA-G gene DNA sequence between mother and child	[76]
		14 bp insertion allele 14 bp + 3142G (insG) haplotype		[75]
		insG/insG diplotype in HCV coinfected		[75]

HCV		insG/insG diplotype in HIV coinfected		[75]
	+3142C allele in sickle cell disease patients			[79]
		−14 bp/−14 bp genotype		[81]
			
	HLA-G*0105N		G*010401 homozygosity for HLA-G 14 bp deletion	[80]

HPV	14 bp ins allele	14 bp del allele del/C haplotype with ICC development		[82]

*Plasmodium falciparum *	+3187G allele and haplotype UTR1	Haplotype UTR3		[83]
	+3010G and +3142C +3010G and +3196G			[49]

**Table 3:** Interference of viral proteins with HLA-G intracellular trafficking; comparison with classical HLA class I molecules.

Virus	Viral protein	Classical HLA class I		HLA-G			References
		Mechanism	Downregulation	Downregulation	No change	Upregulation	
HIV	Nef	Interacts directly with class I domain Redirects to endolysosomal pathway	*↘*		→ truncated cytoplasmic domain		[85]
	Vpu	Redirects to degradation pathway Affects early step in biosynthesis	*↘*	*↘*			[119]

HCMV	US2 US11	Exports for cytosolic degradation	*↘*		→ truncated cytoplasmic domain		[84, 86]
	US3 US6	Retention in endoplasmic reticulum	*↘*	*↘*			[87]
	US10			*↘* cytoplasmic tail			[88]
	pp72 and pp86					*↗*	[99]

Herpes Virus	ICP47	Inhibits TAP (transporter associated with antigen processing)	*↘*	*↘*			[90]
